# Surgically Induced Scleral Necrosis: A Case Report

**DOI:** 10.7759/cureus.83019

**Published:** 2025-04-25

**Authors:** Saad Benchekroun, Narjisse Taouri, Meryem Benchekroun, Adam Tagmouti, Lalla Ouafa Cherkaoui

**Affiliations:** 1 Ophthalmology, Centre Hospitalo-Universitaire Ibn Sina, Hôpital Des Spécialités, Rabat, MAR; 2 Ophthalmology, University Hospital Center, Mohammed V University, Rabat, MAR; 3 Ophthalmology, Centre Hospitalo-Universitaire Ibn Sina, Rabat, MAR; 4 Ophthalmology, Hôpital Des Spécialités, Mohammed V University, Rabat, MAR

**Keywords:** autoimmune disease, ocular surgery, scleromalacia, steroids, surgically induced scleral necrosis

## Abstract

Surgically induced necrotizing scleritis (SINS) is a scleral ulceration that can occur days to years after various ocular surgeries. It is an uncommon complication that may lead to scleral perforation.

We report the case of a 45-year-old diabetic male patient who underwent vitreoretinal surgery on his left eye for the treatment of a complicated diabetic retinal detachment. He presented three months after the procedure with extensive scleromalacia pre-perforans.

This case highlights the risk of SINS following ocular surgery. Therefore, patients at high risk of developing SINS should be identified before undergoing any sclera-involving ocular procedure through thorough ophthalmic and systemic evaluation.

## Introduction

Surgically induced necrotizing scleritis (SINS) is a scleral ulceration that can occur days to years after various ocular surgeries [1,2]. It is a rare and severe complication that may lead to scleral perforation [1]. The pathogenic mechanisms of SINS are still poorly understood; however, contributing factors include trauma, infection, ischemia, toxicity, and autoimmunity. SINS most commonly occurs following pterygium excision or cataract surgery [1].

The main risk factors for SINS include ocular surgery, ocular trauma, use of sutures, corticosteroids, antifibrotic therapies, scleral buckling, cautery, mechanical compression, and autoimmune diseases [1]. SINS can be classified into infectious and non-infectious forms [1]. The treatment of choice for non-infectious SINS includes oral corticosteroids and immunosuppressive therapy [1,3].

We report the case of a 45-year-old male patient who underwent vitreoretinal surgery for a complicated diabetic retinal detachment in his left eye. During follow-up, he presented with extensive scleromalacia pre-perforans. This case highlights the diagnosis and management of this rare complication.

## Case presentation

We report the case of a 45-year-old diabetic man with a history of proliferative diabetic retinopathy in both eyes and macular edema in the right eye. He had been treated with intravitreal anti-VEGF injections and laser photocoagulation. The patient had no history of ocular inflammation or systemic autoimmune disease.

He presented with a complicated diabetic retinal detachment in his left eye, for which he underwent uncomplicated vitreoretinal surgery, including 360-degree endolaser photocoagulation and silicone oil tamponade, without scleral buckling. The patient had well-controlled insulin-dependent diabetes mellitus, both before and after the surgery. His postoperative course was uneventful during the first two weeks, and no scleral ulceration was observed.

**Figure 1:**
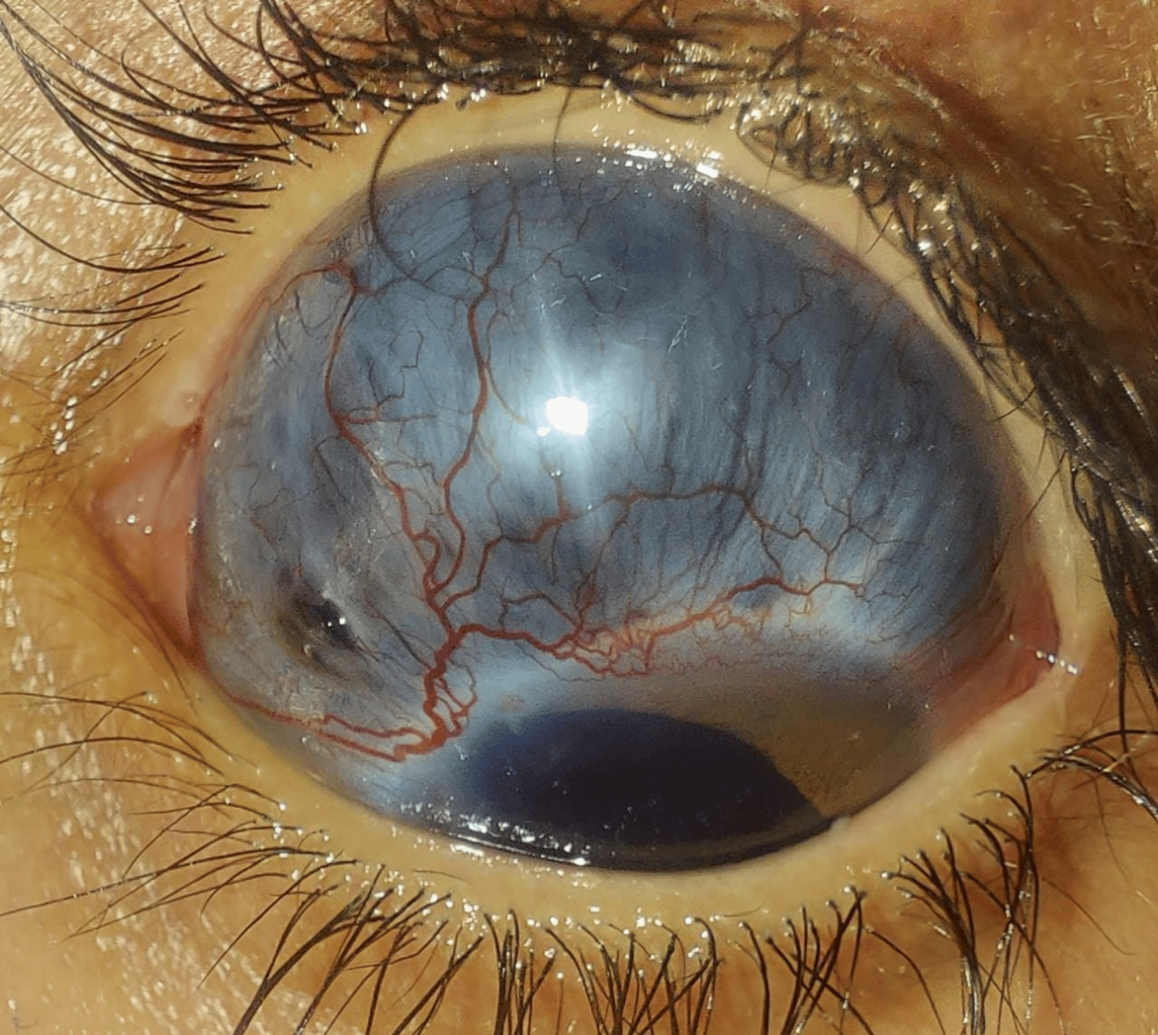
Scleromalacia pre-perforans following vitreoretinal surgery.

At his follow-up visit in the third month, he presented to the emergency department with pain in his left eye but without any change in vision. The visual acuity in that eye was hand motion. Clinical examination revealed severe scleral thinning with imminent choroidal exposure, globe ectasia, a clear cornea, a quiet anterior chamber, corectopia, and a clear lens. Fundus examination showed an attached retina (Figure 1).

The patient underwent a full Work-up evaluation that excluded any associated systemic inflammatory condition or infectious process. The diagnosis of non-infectious SINS was made.

The patient was treated with oral prednisone, with slow taper for two months. The evolution was marked by the stabilization of the scleral thinning without additional treatment. 

## Discussion

SINS is a form of scleral thinning that can occur post-operatively, ranging from day one to more than 50 years [1,2]. This condition is a rare and progressive disease that can lead to scleral perforation [1,3].

To our knowledge, there have been no large studies illustrating the incidence or frequency of SINS. The pathogenesis of this condition remains unclear, and various pathogenic mechanisms have been reported [3,4].

There are case reports following different types of ocular procedures. SINS is most commonly observed in cases of pterygium excision [1,5,6]. Cases of SINS following adjunctive intraoperative therapy, such as antimetabolites, or secondary to excessive cauterization have also been described [1,7-10].

Post-operative scleral necrosis associated with autoimmune diseases, such as rheumatic and chronic degenerative conditions, has also been reported [11,12].

Infectious causes of SINS are most commonly bacterial (Pseudomonas aeruginosa being the most frequent pathogen) [13,5]. Fungal SINS are less common, making an adequate workup essential to differentiate between infectious processes and autoimmune diseases [13-16].

Clinically, most cases of SINS are asymptomatic [1,17]. However, some patients may present with mild pain, conjunctival hyperemia, ciliary injection, and edematous sclera. In more advanced stages, moderate to severe pain, visual loss, peripheral corneal ulcers, staphyloma formation, scleral ulceration, and "porcelain white" necrosis (with or without perforation) may be observed [1,18,19].

When SINS is suspected, the following workup should be performed: eliminating infectious etiologies through cultures and/or scleral biopsy, as well as blood tests including complete blood count, blood chemistry, C-reactive protein, erythrocyte sedimentation rate, RPR and treponemal testing, complement fractions, serum uric acid, anti-citrullinated protein antibodies, rheumatoid factor, anti-nuclear antibodies, anti-neutrophil cytoplasmic antibodies, and purified protein derivative. A chest X-ray and urine analysis should also be done [1]. Regarding medical and surgical management, both local and systemic treatments have been reported [1].

First-line therapy for non-infectious necrotizing scleritis includes topical and oral corticosteroids [1], typically at a dose of 1 mg/kg/day with a weekly taper for a variable period depending on the patient’s response and clinical findings [1,20]. In cases with associated autoimmune diseases, a combination of immunosuppressive agents or biological agents may be necessary to avoid the long-term adverse effects of systemic corticosteroids [1]. For infectious SINS, treatment is based on systemic and topical antibiotics or antifungals, depending on the type of infection [1].

For surgical management of SINS, surgical debridement is recommended in cases of active and progressive scleral necrosis to obtain microbial smear and culture and to reduce local inflammation [1]. Other surgical options include conjunctival and Tenon flaps, autologous fascia lata tectonic grafts, and other tectonic grafts [1].

## Conclusions

SINS is a scleral ulceration that can occur from days to years after various ocular surgeries. Post-operative scleral necrosis can be associated with autoimmune diseases or caused by bacterial or fungal infections. The first-line therapy for non-infectious SINS is corticosteroids. Close postoperative monitoring and early intervention are essential for high-risk patients.
